# Lycopene Alleviates the Adverse Effects of Feeding High-Lipid Diets to Hybrid Grouper (♀*Epinephelus fuscoguttatus* ×♂*E. lanceolatus*)

**DOI:** 10.1155/2023/8814498

**Published:** 2023-10-23

**Authors:** Menglong Zhou, Hao Liu, Baiquan Lu, Biao Li, Weibin Huang, Beiping Tan, Yuanzhi Yang, Xiaohui Dong, Haitao Zhang

**Affiliations:** ^1^Laboratory of Aquatic Nutrition and Feed, College of Fisheries, Guangdong Ocean University, Zhanjiang 524088, China; ^2^Guangdong Engineering Technology Research Center of Aquatic Animals Precision Nutrition and High Efficiency Feed, Zhanjiang, Guangdong 524088, China; ^3^Key Laboratory of Aquatic, Livestock and Poultry Feed Science and Technology in South China, Ministry of Agriculture, Zhanjiang, Guangdong 524000, China

## Abstract

It has been found that high-lipid diets (HLDs) disrupt lipid metabolism in fish, leading to an excessive accumulation of lipids in various tissues of the fish body. The objective of this study was to investigate if the inclusion of lycopene (LCP) in an HLD may mitigate the adverse consequences of excessive dietary lipid intake in hybrid grouper (♀ *Epinephelus fuscoguttatus* × ♂ *E. lanceolatus*). The experimental design incorporated a control group (L0), which was administered a diet consisting of 42% protein and 16% lipid. The diets for groups L1, L2, and L3 were developed by augmenting the control diet with 100, 200, and 400 mg/kg LCP, respectively. The duration of the trial spanned a period of 42 days. The results of the study showed that the weight gain rate (WGR) and protein efficiency ratio (PER) of the three LCP treatment groups (L1, L2, and L3) tended to increase and then decrease, with a significant increase in WGR and PER in L2 (*P*  < 0.05). Visceral somatic index and hepatic somatic index tended to decrease and then increase in all treatment groups, with a significant decrease in L2 (*P*  < 0.05). In serum dietary LCP significantly reduced triglyceride (TG), total cholesterol (TC), and low-density lipoprotein (LDL) content and significantly increased high-density lipoprotein (HDL) content (*P*  < 0.05). In the liver, dietary LCP reduced TC, TG, and very LDL levels and improved lipoprotein lipase, hepatic lipase, fatty acid (FA) synthetase, and acetyl-CoA carboxylase activities. The number and area of hepatic lipid droplets decreased significantly with increasing LCP content. In the liver, the addition of appropriate levels of LCP significantly upregulated lipoprotein lipase (*lpl*) and peroxisome proliferator-activated receptor *α* (*pparα*). In summary, dietary LCP improves growth and reduces lipid deposition in the liver of hybrid grouper by increasing lipolytic metabolism and decreasing FA synthesis. Under the experimental conditions, the fitted curve analysis showed that the recommended LCP additions to the high lipid diet for juvenile hybrid grouper were 200–300 mg/kg.

## 1. Introduction

Marine species known as hybrid groupers (♀*Epinephelus fuscoguttatus* × ♂*Epinephelus lanceolatus*) have a great flavor, a high economic value, and a high nutritional value [1, 2]. According to studies, hybrid groupers perform better in terms of development, stress resilience, and economic value than their purebred parents [3, 4]. At dietary lipid levels of 7% (45%–55% of dietary protein), hybrid grouper had the highest protein efficiency ratio (PER), according to the study. Higher levels of dietary lipids (10%, 13%) decrease PER while increasing abdominal lipid deposition [5]. Other studies showed that higher levels of dietary lipids (16%) were significantly accelerated the growth rate of grouper while significantly increasing the crude lipid content of the fish in all tissues, suggesting that high-lipid diets lead to excessive lipid deposition in all tissues of grouper [6]. A high-lipid diet (HLD) with a lipid level of 16% and protein level of 42% was used in this study.

Dietary lipids and oils have a high-energy density that can provide energy as well as important compounds, including vital fatty acids (FAs), lipid-soluble vitamins, phospholipids, and sterols for sustaining life and maintaining appropriate cell structure and biological function [7]. Fish that are fed an HLD have better protein utilization rates, higher diet efficiency, and lower dietary costs [8, 9]. Therefore, aquaculture uses it frequently [10–12]. However, most studies have shown that high levels of lipid intake can lead to abnormal accumulation of lipids in various tissues of the fish body and cause damage to the fish liver, thus affecting the health of the fish [13, 14]. In fish, the primary harm of fatty liver included degeneration and necrosis of hepatic cells and a decrease in stress resistance [15, 16]. Thus, it is important to identify meaningful strategies to mitigate the adverse impacts of HLD.

In recent years, a considerable body of study has demonstrated the potential of many plant extracts, such as silymarin, tea polyphenols, *Panax notoginseng*, and ginkgo biloba leaf, to positively impact fish development, liver lipid metabolism, and immune function [15–19]. This observation demonstrates that the utilization of plant extract as an environmentally friendly and effective addition holds significant promise for its application in the field of aquaculture. Lycopene (LCP), which belongs to the carotenoid family, is predominantly present in tomatoes, melons, and carrots. It possesses antioxidant properties, exhibits anti-inflammatory effects, and is capable of regulating lipid metabolism [20]. Several studies have indicated that the incorporation of LCP into the diet of goldfish (*Carassius auratus*) and rainbow trout (*Oncorhynchus mykiss*) not only promotes development but also enhances feed utilization and antioxidant capacity [21, 22]. The utilization of LCP has been shown to be an effective strategy in mitigating the toxicity associated with its exposure in Nile tilapia (*Oreochromis niloticus*) [23]. LCP supplementation in HLD-fed rats (*Rattus norvegicus*) decreased hepatic lipid synthesis and lipid levels in both mother and progeny mice [24]. LCP was discovered in a study on nonalcoholic fatty liver disease to increase the expression of *pparα* [25]. However, there are few studies on the application of LCP in HLD.

In order to increase production and reduce costs, most commercial diets for this species are HLD. It has been found that HLD leads to excessive lipid deposition in various tissues of the fish body, affecting the body's ability to resist disease and reducing the quality of the product [20, 26]. Numerous studies have been done on reducing the negative effects of HLD-fed hybrid groupers. However, studies on the negative effects of LCP supplementation in HLD of hybrid grouper to reduce HLD have not been conducted. This investigation aimed at estimating the effect of dietary LCP levels on growth and lipid metabolism in hybrid grouper-fed HLD.

## 2. Materials and Methods

The National Institutes of Health's “Guide for the Care and Use of Laboratory Animals” was strictly followed when conducting all animal experiments. The Guangdong Ocean University's Animal Ethics Committee (Zhanjiang, China) gave its approval to the animal protocols (approval ID: GDOU-IACUC-2022-A0502; approval date: May 2, 2022).

### 2.1. Experimental Diets

Guangdong Yuejia Feed Co., Ltd. provides the raw materials. From Shandong Shengjiade Biotechnology Co. in Shandong, China, LCP (10%) was purchased. The primary protein sources were red fish meal, soybean protein concentrate, and soybean meal, while the primary lipid sources were fish oil and corn oil (Table 1). Five different diets were prepared: a diet with 42% protein and 16% lipids for the control group (L0). Then, the negative control diet was modified by adding 100, 200, and 400 mg/kg of LCP to create the experimental diets for L1, L2, and L3. All feed ingredients are accurately weighed in accordance with the proportions of the feed formula, sieved through a 60-mesh sieve, and then mixed evenly using the step-by-step premixing method [27]. Fish oil, corn oil, and soy lecithin were mixed and added to the mixed feed ingredients. They were then mixed again and passed through a 40-mesh sieve. The mixed raw materials were processed into 2.0 mm diameter pellets (extruding temperature: 85–90°C) by a twin-screw extruder (F–26, South China University of Technology, Guangdong Province, China). The diets are then bagged, sealed, and kept in a refrigerator at −20°C after air drying (the diets are spread out and allowed to dry by themselves).

### 2.2. Fish and Feeding Trial

A feeding experiment was conducted in the running water culture system of Donghai Island Hengxing South Science and Technology Co., Ltd. (Zhanjiang, China). On Donghai Island (Zhanjiang, China), a local hatchery offers juvenile hybrid grouper. The young fish were domesticated with commercial diets and briefly raised in 1 m^3^ buckets prior to the experiment. The temporary period's management of the environment and culture was identical to that of the formal experiment. Before the experiment started, 180 healthy and disease-free grouper juveniles (20.56 ± 0.17 g) were randomly divided into 15 plastic tanks (100 L), with three replicates of each feed and 15 fish per replicate, and running water was turned on for the culture experiment. Until they appeared to be satisfied, groupers were fed twice daily (7:30–8:30 and 16:30–17:30). Daily records were kept of the number of dead fish and the amount of food consumed. During the experiment, the water's environmental conditions were 27–31°C, pH 7.6–7.8, salinity 26–28, and dissolved oxygen >7 mg/L. The 42-day experiment was conducted.

### 2.3. Sample Collection

All experimental fish fasted for 24 hr prior to sample collection [28]. Keep track of how many and how much fish are in each bucket overall (anesthetized with eugenol (1 : 10,000)). Blood from four fish from each tank is drawn with a 1 ml sterile syringe into 1.5 ml microcentrifuge tubes and allowed to stand for 12 hr at 4°C, then centrifuged at 4°C (3,000 rpm for 10 min) to collect serum, which should be stored at −80°C immediately prior to analysis. Additionally, measurements of three fish's morphological indices (such as body weight, length, liver weight, visceral mass, weight, etc.) were made. Livers were taken (per tank) at random from three of the four fish. The liver and visceral masses were precisely weighed for testing purposes. The appropriate ratio of 1 : 9 (weight of liver (g): volume of normal saline (mL)) was used to homogenize the liver and normal saline in an ice bath. After mechanical homogenization in an ice-water bath, it was centrifuged at 2,500 rpm for 10 min (4°C) to collect supernatant for detection of enzyme activities. About 50 g of muscle per tank for muscle proximate composition analysis and two whole fish were collected. In each bucket, the livers of three additional fish were removed. Some of the livers were loaded into centrifuge tubes containing 4% paraformaldehyde to create oil-red slices, and the remaining livers were later cut into 2 mL enzyme-free centrifuge tubes containing RNA. The livers were moved to −80°C and left there for 12 hr while RNA was soaked into the liver tissue for later analysis.

### 2.4. Methods of Analysis

Partial abbreviation: IBW (g): initial mean body weight; FBW(g): final mean body weight; NFL: number of fish left per bucket; NF: number of fish per bucket; AWG (g): average weight gain; API (g): average protein intake; LW (g): live weight; BL (cm): body length; BW (g): body weight; VW (g): visceral weight. Calculation formula of growth performance: SR (survival rate, %) = 100% × (NFL/NF); WGR (weight gain rate, %) = 100 × [(FBW, g) – (IBW, g)]/(IBW, g); PER (protein efficiency ratio, %) = 100 × (AWG, g)/(API, g). Morphological index calculation: CF (condition factor, g/cm^3^) = 100 × LW (g)/(BL, cm)^3^; HSI (hepatic somatic index, %) = 100 × LW (g)/BW (g); VSI (visceral somatic index, %) = 100 × VW (g)/BW (g).

Indicators of enzyme activity measured in serum include total cholesterol (TC), triglycerides (TGs), low-density lipoprotein (LDL), and high-density lipoprotein (HDL) levels. Indicators of enzyme activity measured in the liver included lipoprotein lipase (LPL), fatty acid synthase (FAS), acetyl coenzyme (ACC), and hepatic lipase (HL) activities, as well as very low-density lipoprotein (VLDL), TC, and TG levels. All enzyme activity indicators were analyzed using commercial ELISA kits (Shanghai Enzyme Link Biotechnology Co., Ltd., Shanghai, China). The specific assay procedure was carried out strictly according to the steps in the instructions.

### 2.5. RNA Extraction and cDNA Synthesis

Total RNA was extracted by TRIzol (genetically modified Biotechnology, Beijing, China). The specific assay procedure was carried out strictly according to the steps in the instructions. A NanoDrop2000 spectrophotometer (Guangzhou Genome Co., Ltd., China) was used to detect the concentration and quality of RNA at 260 and 280 nm. The integrity of total RNA was verified by 1% agarose gel electrophoresis. CDNA was synthesized by reverse transcription with the AG kit (Hunan Accurate Biological Engineering Co., Ltd., Changsha, China). Stored at −80°C, it was retained for real-time quantitative polymerase chain reaction (RT-qPCR).

### 2.6. RT-qPCR

The primer templates of the reference gene (*β-actin*) and the target gene (*fas*, *pparα*, and *lpl*) were designed based on the published sequence of the grouper genes (Table 2). The RT-qPCR was carried out on a 384-well plate with a reaction volume of 10 *μ*L, including 5 *μ*L of SYBR® Green RT-qPCR Master Mix, 0.8 *μ*L of each primer template, 1 *μ*L of cDNA template, and 3.2 *μ*L RNase-free water. Under the conditions of incubation at 95°C for 30 s, 95°C for 5 s, and 60°C for 34 s, the gene expression level of each sample was detected by the RT-qPCR method, and the threshold cycle (Ct value) was collected. The 2^−*ΔΔ*Ct^ method is used to calculate the relative expression level of the target gene [16, 29].

### 2.7. Oil Red O (ORO) Staining

ORO staining was used to quantify the amount of lipid deposition in the liver. Frozen section preparation (Servicebio, China) (Tissue fixation; dehydration; optimal cutting temperature embedding; frozen sectioning) The embedding stage was fixed on a microtome, and the surface of the tissue was roughly trimmed first, and then slicing was begun with a slice thickness of 8–10 *μ*m. Clean slides were placed flat on the tissue sections, and the tissue was secured to the slides. Staining: sections are stained with oil-saturated O for 8–10 min (protected from light and covered). Color separation: color separation used 60% isopropyl alcohol. Washing: the slide was dipped into distilled water with gentle movements to prevent displacement of the lipid droplets. Sealing: dried excess water with filter paper, then sealed the piece with glycerin. Finally, the lipid droplets (LDs) were observed with a light microscope (Nikon Eclipse, Japan) at 400x magnification.

### 2.8. Statistical Analysis

All results were tested by chi-square using SPSS 22.0 (SPSS Inc., USA) and expressed as mean ± standard error. One-way analysis of variance and Tukey's multiple extreme difference test were used to determine significant differences between treatments. ORO staining results were analyzed using Image Pro Plus 6.0. Probability values of *P*  < 0.05 being statistically significant.

## 3. Results

### 3.1. Growth and Morphological

**Figure 1:**
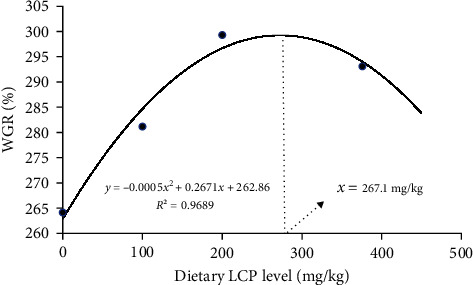
Fitted curve analysis of the relationship between dietary LCP levels and weight gain rate (WGR) of hybrid grouper. The appropriate level of LCP in HLD was 267.1 mg/kg.

Table 3 shows that FBW, WGR, and PER were significantly higher in L2 than in L0 (*P*  < 0.05). The fitted curve analysis based on WGR (Figure 1) showed that 267.1 mg/kg dietary LCP was recommended in hybrid grouper HLD. The hybrid grouper's SR was unaffected by the addition of LCP to the HLD (*P*  > 0.05). CF increases with increasing LCP content, with a maximum at L3 (*P*  < 0.05). However, there was no significant difference with L0 (*P*  > 0.05). As the LCP content increases, HSI and VSI decrease and then increase, with a minimum at L2, and it is significantly lower than L0 (*P*  < 0.05).

### 3.2. Whole Body and Muscle Composition

As shown in Table 4, with the increase in dietary LCP content, the whole-body moisture content and crude protein content of the LCP treatment group tended to increase and then decrease, and the whole-body crude lipid content tended to gradually increase. However, there was no difference with L0 (*P*  > 0.05). In the HLD group, the addition of LCP had no effect on muscle moisture. Muscle crude protein levels were higher in all LCP-treated groups than in the L0 (*P*  < 0.05). The addition of LCP reduced muscle crude lipid, but there was no significant difference from L0 (*P*  > 0.05).

### 3.3. Serum Biochemical Index

**Figure 2:**
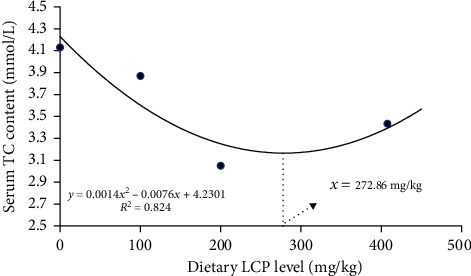
Fitted curve analysis of the relationship between dietary LCP levels and serum TC of hybrid grouper. The appropriate level of LCP in HLD was 272.86 mg/kg.

As shown in Table 5, the levels of TG were not significantly different (*P*  > 0.05). LCP-treated groups all had significantly lower serum TC levels than L0 (*P*  < 0.05). HDL levels increased with increasing LCP levels and were greatest at L3 (*P*  < 0.05). LDL levels increased and then decreased with increasing LCP levels and were greatest at L2, and were all significantly lower than L0 (*P*  < 0.05). The fitted curve analysis based on serum TC (Figure 2) showed that the optimum dietary LCP level was 272.86 mg/kg.

### 3.4. Biochemical Indexes and Activities of Enzymes Related to Lipid Metabolism in the Liver

Table 6 shows that the TC levels of all LCP-treated groups were significantly lower than those in the L0 (*P*  < 0.05). TG increased with increasing LCP levels, and only the TG level in L3 was significantly lower than that in L0 (*P*  < 0.05). With the rise in LCP level, VLDL content and FAS activity steadily dropped, and the lowest values were reported in L3, which were substantially lower than those in L0 (*P*  < 0.05). Compared with L0, only L3 substantially increased LPL activity in the LCP treatment group (*P*  < 0.05). Liver HL levels tended to increase with increasing dietary LCP levels but were not significantly different from L0 (*P*  > 0.05). ACC levels were considerably lower in all LCP treatment groups than those in L0, with the lowest value in L2 (*P*  < 0.05).

### 3.5. ORO Staining

**Figure 3:**
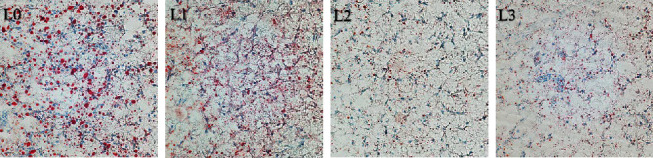
Oil red O slices of liver tissue of hybrid grouper fed with different diets (slice oil red × 400). Quantifying lipid accumulation by measuring the number and area of stained oil droplets. Values were presented as means ± SE of three replicates, and mean values with unlike letters indicate significant differences (*P*  < 0.05).

The results of oil red-stained liver sections are shown in Figure 3. In oil red staining, nuclei and lipids are dyed blue and red, respectively. With the increase in LCP levels, the number and area of lipid droplets in the liver decreased significantly (*P*  < 0.05).

### 3.6. Expression of Lipid Metabolism-Related Genes in the Liver

**Figure 4:**
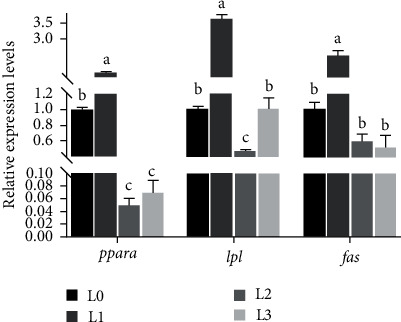
Expression of genes related to lipid metabolism in the liver of hybrid groupers fed with different diets. Values are mean ± SE (*n* = 3). *fas*: fatty acid synthase; *lpl*: lipoprotein lipase; *pparα*: peroxisome proliferator-activated receptor *α*.

As indicated in Figure 4. The expression levels of *pparα* and *lpl* first decreased and then increased with increasing LCP levels and were greatest in L1, and both were significantly higher than in L0 (*P*  < 0.05). Whereas the expression level of *fas* gradually decreased with the increase of LCP level and had a minimum at L3, but did not differ from that of L0 (*P*  > 0.05).

## 4. Discussion

Previous studies have shown that 7% dietary lipid at 45%–55% dietary protein levels increased PER and thus improved grouper growth performance, but higher dietary lipids (10% and 13%) significantly reduced PER and elevated intra-abdominal lipid ratios, leading to lipid deposition in various tissues of the fish [5]. Another study showed that 50% protein and 14% lipid [30] or 44% protein and 16% lipid [6] can promote the growth of hybrid groupers. Therefore, HLD (42% protein and 16% lipid) was used in this study to maintain higher PER and WGR. In crucian carp (*Cyprinus carpio*) [22], the addition of LCP increased WGR, whereas, in rainbow trout [31], supplementation of the diet with 0.02% LCP increased PER; and in rainbow trout [32], growth increased with increasing LCP additions. In this study, the weight gain rate of hybrid grouper rose with increasing LCP content in the diet; the addition of an adequate concentration of LCP (267.1 mg/kg) to HLD could considerably raise the FBW and WGR of hybrid grouper.

The influence of dietary lipid levels on HSI, VSI, and CF in orange-spotted grouper (*Epinephelus coioides*) [33] and silver barb (*Barbonymus gonionotus*) [34] has been explored. Some studies have demonstrated that the rise of HSI, VSI, and CF in HLD is connected to lipid accumulation [35–37]. It has been shown that the dietary addition of LCP reduced lipid deposition in rats [38] fed an HLD and the abdominal lipid rate of broilers [39]. In the present study, CF in all LCP treatment groups did not differ from that in the L0 treatment group. VSI was significantly lower than L0 in all LCP treatment groups except L3. HSI was significantly lower than L0 in all LCP addition groups. Muscle crude protein and crude lipid contents were similarly lower in the LCP treatment groups.

Blood lipids contain TGs, TC, phospholipids, and free FAs [40]. It has been shown that feeding HLD leads to increased serum TC, TG, and LDL levels and decreased HDL levels [31, 41]. The variations in HDL and LDL revealed the problem of lipid metabolism [16]. HDL transfers cholesterol from extrahepatic tissue to the liver for processing in the form of HDL-C. LDL transfers endogenous cholesterol to extrahepatic tissue in the form of LDL cholesterol. The results of this study suggest that dietary LCP increases HDL levels and promotes the metabolism of serum TG and TC. This is similar to the findings of prior studies on rats [24]. Disorders of lipid metabolism include increased serum levels of TC and TG and decreased levels of HDL [42]. Relatively low levels of TC reflect good lipid metabolism, whereas relatively high levels of HDL are characteristic of a healthy body [43]. Therefore, serum TC and HDL levels are the basis for dietary inositol to promote lipid metabolism in grouper. The optimum level of dietary LCP addition was found to be 272.86 mg/kg by fitting curve based on serum TC.

The liver is the key organ for fish FA-*β*-oxidation and lipid production. Therefore, the modulation of liver lipid production or degrading enzymes in the linked metabolic pathways plays a crucial role in lipid metabolism. LPL and HL in fish liver are the key enzymes of lipid degradation. LPL is a critical enzyme that catalyzes the degradation of lipid particles such as TC, TG, and VLDL [44]. Therefore, the activity of LPL in different tissues can be used to evaluate the decomposition, metabolism, or storage of dietary lipids in the body [45]. HL may enhance the entrance of VLDL into hepatocytes and directly contribute to the reverse transport of HDL. HL may breakdown TGs and create free FAs to lower the level of hepatic lipids [46, 47]. Reports have revealed that an increase in HL and LPL activity increases lipid breakdown metabolism in the liver [48, 49]. FAS, an enzyme that disrupts the reaction of acetyl-CoA, propionyl-CoA, and NADPH, participates in the ab initio synthesis of long-chain FAs [50]. This has also been confirmed in the study of Spotted seabass (*Lateolabrax maculatus*) [51]. ACC is a rate-limiting enzyme for *de novo* synthesis and oxidation of FAs [52]. The findings demonstrated that HLD might enhance the lipid deposition of hybrid groupers and induce abnormalities in lipid metabolism. This is similar to the findings in American black bass (*Micropterus Salmoides*) [53] and Nile tilapia [54]. The addition of LCP to HLD might enhance hepatic lipid metabolism in mice [25, 55]. Supplementing maternal meals with LCP may diminish the activity of ACC and FAS in the liver and lower blood lipid levels in rats and their offspring [24]. In the investigation of broilers, it was observed that LCP could lower the level of blood lipids and the activity of FAS and ACC in the liver [39]. In this trial, the addition of LCP to the ration dramatically lowered hepatic TC and VLDL levels to levels comparable to those of the normal lipid group, demonstrating that LCP may ameliorate the detrimental effects of HLD. Dietary LCP triggers lipid metabolism by upregulating LPL and HL. Correspondingly, the findings on body composition suggested that lipid formation was decreased by LCP in the ration. With the rise of LCP addition, LPL and HL activities in each treatment L3 exhibited a rising trend and were maximal in L2. This shows that LCP may minimize lipid deposition by enhancing lipolysis-related enzyme activity and lowering lipid synthesis-related enzyme activity. From the data, we reasoned that LCP in the food increased lipolysis (LPL, HL) and inhibited lipid synthesis (FAS, ACC), which resulted in the rate of lipolysis being larger than the rate of lipid synthesis and, hence, decreased lipid deposition in the body composition. This is consistent with the results of a number of earlier studies [16].

Excessive accumulation of lipids is an important sign of many metabolic diseases [56, 57]. ORO staining is a useful tool for tissue pathology diagnosis and can assess the degree of lipid degeneration [58]. ORO staining sections can be used to observe the amount of lipids in cells by staining lipid droplets [59]. The morphology of LD can be stained with ORO, and ORO staining has been shown to be effective in visualizing the underlying changes occurring in tissues during the onset and progression of metabolic diseases [52]. In the present study, LCP in the ration significantly attenuated HLD-induced hepatic lipid deposition, and computerized analyses showed that dietary LCP supplementation was effective in reducing the number and area of LD. This shows that the addition of suitable LCP can attenuate HLD-induced lipid deposition. This is also in agreement with the results of liver enzyme activities. This indicates that the addition of LCP can promote lipid metabolism and inhibit lipid synthesis to reduce HLD-induced lipid deposition. However, there was a slight increase in the number and volume of hepatic LDs in L1 compared to L2. This may be related to the amount of LCP added. It needs to be further explored in conjunction with other results.

To explore the mechanism of lipid metabolism in hybrid grouper, we analyzed the expression of some of the important liver genes involved in lipid metabolism (lipolysis: *pparα*, *lpl*; lipogenesis: *fas*) with reference to previous studies [16]. It is reported that LCP can improve lipid metabolism in rats [60]. PPAR*α* is activated by FAs as a nuclear receptor and plays a role in FA oxidation [61]. Liver *pparα* plays a role by stimulating the transcription of FA oxidation-related genes [62]. It has been reported that the deficiency of *pparα* in hepatocytes can destroy the dynamic balance of lipid metabolism in the liver and lead to lipid accumulation [63]. In vitro, it was found that LCP significantly inhibited lipid accumulation in HepG2 hepatocytes treated with palmitate by stimulating the expression of *pparα*, thus promoting lipolysis. LPL has been demonstrated to play a vital role on the TG in plasm lipoproteins to release FAs, which are transported to other organs or the adipose tissue for storage [64]. It has been shown that in the hybrid grouper, feeding HLD significantly reduced *lpl* expression [65]. In this study, appropriate LCP supplementation in the ration significantly upregulated the lipolytic genes *pparα* and *lpl*. This result suggests that LCP may reduce lipid deposition by promoting the upregulation of lipolytic genes. The lipid-lowering results of dietary LCP were in agreement with the findings of Arbor Acres Broilers [38].

Interestingly, adipogenic genes (*fas*) were first upregulated and then downregulated with increasing concentrations of dietary LCP, suggesting that a small amount of LCP supplementation also positively affects adipogenesis. In contrast, a large amount of LCP supplementation suppresses adipogenesis. Our present results also indicate that appropriate LCP supplementation in the diet showed some inhibition of lipid deposition in the whole body, muscle, and liver. Similarly, LCP supplementation in HLD downregulated *fas* expression, as reported in mice (*Mus musculus*) [20] and Broiler chickens [39] studies. These results suggest that LCP may reduce lipid deposition in the liver of hybrid groupers fed with HLD by inhibiting lipid anabolism and promoting lipid catabolism.

## 5. Conclusion

The present study showed that dietary LCP can improve growth performance and promote lipid metabolism by decreasing fatty acid synthesis and increasing lipolysis. Thus, dietary LCP alleviated hepatic lipid deposition induced by HLD. Fitted curve analysis based on WGR and serum TC indicated that the appropriate amount of LCP added to the high lipid diet of juvenile hybrid grouper was 200–300 mg/kg.

## Figures and Tables

**Table 1:** Composition and nutrient levels of experimental diets (g/kg).

Ingredient	Diets			
	L0	L1	L2	L3
Fish meal	360	360	360	360
Wheat gluten	50	50	50	50
Wheat flour	170	170	170	170
Soybean meal	100	100	100	100
Soybean protein concentrate	100	100	100	100
Soybean lecithin	15	15	15	15
Fish oil	50	50	50	50
Corn oil	65	65	65	65
Premix^a^	10	10	10	10
Vitamin C (35%)	0.5	0.5	0.5	0.5
Choline chloride (98%)	5	5	5	5
Calcium dihydrogen phosphate	15	15	15	15
Antioxidant^b^	1	1	1	1
Attractant^c^	1.5	1.5	1.5	1.5
Carboxymethylcellulose sodium	20	20	20	20
Corn starch	37	36.9	36.8	36.6
LCP (10%)	0	0.1	0.2	0.4
Total	1,000	1,000	1,000	1,000
Proximate composition^d^				
Moisture (%)	9.15	8.11	9.37	8.43
Crude protein (%)	42.07	42.06	41.94	42.05
Crude lipid (%)	15.78	15.90	15.74	15.72

^a^Vitamin and mineral premixes (IU or g/kg): vitamin A ≥ 45 × 105, vitamin D3 ≥ 12 × 105, Dl-a-tocopherol acetate ≥ 40 g, vitamin K3 ≥ 8 g, vitamin B1 ≥ 6 g, vitamin B2 ≥ 9 g, vitamin B6 ≥ 7 g, vitamin B12 ≥ 0.05 g, D-calcium pantothenate ≥ 30 g, nicotinamide ≥ 45 g, folic acid ≥ 2.5 g, D-biotin ≥ 0.1 g, inositol ≥ 100 g, provided by Qingdao Master Biotechnology Co., Ltd. (Qingdao, China). ^b^Antioxidant: ethoxyquin. ^c^Attractant composition: taurine: glycine: betaine = 1 : 3 : 3. ^d^Measured value.

**Table 2:** Primers used in RT-qPCR.

Primers names	Forward and reverse primers sequence (5–3′)	Genbank accession no.
*β-actin*-F/R	GGCTACTCCTTCACCACCACA TCTGGGCAACGGAACCTCT	XM_033645256.1

*pparα*-F/R	CATCGACAATGACGCCCTC GCCGCTATCCCGTAAACAAC	XM_049574358.1

*lpl*-F/R	AGAAGACAACATGAGCCGTAAA AGAGTTGTTCTGCCCGTAAAG	XM_049587374.1

*fas*-F/R	GGCAGAGAGGACAACAGTAAAG GTGTCAGGGTTCAGGCTATTT	XM_049563855.1

fas, fatty acid synthase; lpl, lipoprotein lipase; ppar*α*, peroxisome proliferator-activated receptor *α*.

**Table 3:** Growth performance and morphological indexes of hybrid groupers fed with different diets.

Items	Diets			
	L0	L1	L2	L3
IBW (g)	20.61 ± 0.02	20.62 ± 0.03	20.56 ± 0.03	20.59 ± 0.03
FBW (g)	85.56 ± 2.23^b^	88.80 ± 1.40^ab^	92.27 ± 0.98^a^	90.74 ± 0.87^ab^
WGR (%)	264.18 ± 9.26^b^	281.16 ± 6.89^ab^	299.31 ± 4.86^a^	290.82 ± 5.43^ab^
SR (%)	96.67 ± 3.33	96.67 ± 3.33	100 ± 0.00	93.33 ± 6.67
PER (%)	2.83 ± 0.07^b^	2.88 ± 0.04^b^	2.95 ± 0.06^a^	2.83 ± 0.08^b^
CF (g/cm^3^)	2.90 ± 0.11^ab^	2.74 ± 0.06^b^	2.79 ± 0.06^b^	3.07 ± 0.06^a^
VSI (%)	15.20 ± 0.27^a^	14.32 ± 0.30^b^	12.55 ± 0.25^c^	14.66 ± 0.14^ab^
HSI (%)	5.42 ± 0.32^a^	4.74 ± 0.19^b^	4.12 ± 0.23^b^	4.68 ± 0.21^b^

Values are means ± SE (*n* = 3). Data with different superscript letters in the same row are statistically significant (*P*  < 0.05).

**Table 4:** Whole body and muscle composition (wet weight %) of hybrid groupers fed with different diets.

Items	Diets			
	L0	L1	L2	L3
Whole body				
Moisture (%)	67.30 ± 0.19	68.14 ± 0.20	68.47 ± 0.53	67.91 ± 0.21
Crude protein (%)	17.49 ± 0.43	18.18 ± 0.01	19.23 ± 0.33	17.75 ± 0.23
Crude lipid (%)	6.33 ± 0.48	7.08 ± 0.16	6.98 ± 0.27	7.69 ± 0.10
Muscle				
Moisture (%)	78.65 ± 0.25	78.36 ± 0.36	78.20 ± 0.32	78.46 ± 0.4
Crude protein (%)	17.90 ± 0.10^b^	19.47 ± 0.38^a^	19.63 ± 0.14^a^	19.22 ± 0.08^a^
Crude lipid (%)	1.51 ± 0.05	1.30 ± 0.04	1.29 ± 0.10	1.30 ± 0.10

Data in the table are expressed as mean ± SE (*n* = 3). Data with different superscript letters in the same row are statistically significant (*P*  < 0.05).

**Table 5:** Serum biochemical indexes of hybrid groupers fed with different diets.

Items	Diets			
	L0	L1	L2	L3
TC (mmol/L)	4.13 ± 0.17^a^	3.87 ± 0.05^b^	3.05 ± 0.08^c^	3.4 ± 0.03^c^
TG (mmol/L)	1.31 ± 0.02^ab^	1.12 ± 0.08^ab^	1.09 ± 0.05^b^	1.47 ± 0.07^a^
HDL (mg/L)	34.83 ± 0.40^d^	40.50 ± 0.69^c^	46.13 ± 0.09^b^	52.77 ± 0.90^a^
LDL (mmol/L)	5.62 ± 0.07^a^	4.26 ± 0.07^c^	5.33 ± 0.02^b^	3.74 ± 0.09^d^

Data in the table are expressed as mean ± SE (*n* = 3). Differences with different superscript letters in the same row are statistically significant (*P*  < 0.05). TG, triglycerides; TC, total cholesterol; HDL, high-density lipoprotein; LDL, low-density lipoprotein.

**Table 6:** Liver biochemical indexes and lipid metabolism enzymes activities of hybrid groupers fed with different diets.

Items	Diets			
TC (*μ*mol/mg.pro)	7.60 ± 0.33^a^	6.00 ± 0.22^c^	6.51 ± 0.24^bc^	4.72 ± 0.42^d^
TG (*μ*mol/mg.pro)	1.47 ± 0.08^a^	1.34 ± 0.03^a^	1.31 ± 0.02^a^	1.06 ± 0.07^b^
VLDL (*μ*mol/mg.pro)	17.75 ± 0.10^a^	17.10 ± 0.83^ab^	15.73 ± 0.35^b^	12.04 ± 0.74^c^
LPL (mU/mg.pro)	596.55 ± 13.67^b^	616.35 ± 44.61^b^	646.82 ± 34.87^b^	751.44 ± 41.26^a^
HL (U/mg.pro)	36.28 ± 2.29	37.21 ± 1.45	37.34 ± 1.59	40.90 ± 0.51
FAS (mU/mg.pro)	2,602.39 ± 26.43^a^	2,164.30 ± 32.04^b^	2,033.96 ± 28.33^bc^	1,740.15 ± 75.68^d^
ACC (mU/mg.pro)	54.43 ± 2.47^a^	44.07 ± 0.9^b^	34.74 ± 1.58^c^	44.55 ± 0.86^b^

Data in the table is represented by average value ± SE (*n* = 3). The significant difference is exhibited in the same row with different superscript letters (*P*  < 0.05). LPL, lipoprotein lipase; HL, hepatic lipase; FAS, fatty acid synthetase; VLDL, very low-density lipoprotein; TG, triglyceride; ACC, acetyl-CoA carboxylase and TC, total cholesterol.

## Data Availability

The data used to support the findings of this study are available from the corresponding author upon request.
